# Distribution and elimination kinetics of midazolam and metabolites after post-resuscitation care: a prospective observational study

**DOI:** 10.1038/s41598-024-54968-z

**Published:** 2024-02-25

**Authors:** Wonjoon Jeong, Jung Sunwoo, Yeonho You, Jung Soo Park, Jin Hong Min, Yong Nam In, Hong Joon Ahn, So Young Jeon, Jang Hee Hong, Ji Hye Song, Hyein Kang, My Tuyen Thi Nguyen, Jaehan Kim, Changshin Kang

**Affiliations:** 1 Department of Emergency Medicine, Chungnam National University Hospital, Daejeon, 35015 Republic of Korea; 2 Department of Emergency Medicine, College of Medicine, Chungnam National University, Daejeon, 35015 Republic of Korea; 3 Clinical Trials Center, Chungnam National University Hospital, Daejeon, 35015 Republic of Korea; 4 Department of Emergency Medicine, Sejong Chungnam National University Hospital, Sejong, 30099 Republic of Korea; 5 Department of Pharmacology, College of Medicine, Chungnam National University, Daejeon, 35015 Republic of Korea; 6 Department of Medical Science, College of Medicine, Chungnam National University, Daejeon, 35015 Republic of Korea; 7 Department of Food and Nutrition, Chungnam National University, Daejeon, 34134 Republic of Korea; 8 Department of Food Technology, Can Tho University, Can Tho City, 90000 Vietnam

**Keywords:** Out-of-hospital cardiac arrest, Midazolam, Pharmacokinetics, Sedation, Brain injuries, Hypoxic-ischaemic encephalopathy

## Abstract

Administration of sedatives for post-resuscitation care can complicate the determination of the optimal timing to avoid inappropriate, pessimistic prognostications. This prospective study aimed to investigate the distribution and elimination kinetics of midazolam (MDZ) and its metabolites, and their association with awakening time. The concentrations of MDZ and its seven metabolites were measured immediately and at 4, 8, 12, and 24 h after the discontinuation of MDZ infusion, using liquid chromatography-tandem mass spectrometry. The area under the time-plasma concentration curve from 0 to 24 h after MDZ discontinuation (AUC_last_) was calculated based on the trapezoidal rule. Of the 15 enrolled patients, seven awakened after the discontinuation of MDZ infusion. MDZ and three of its metabolites were major compounds and their elimination kinetics followed a first-order elimination profile. In the multivariable analysis, only MDZ was associated with awakening time (AUC_last_: R^2^ = 0.59, *p* = 0.03; AUC_inf_: R^2^ = 0.96, *p* < 0.001). Specifically, a 0.001% increase in MDZ AUC was associated with a 1% increase in awakening time. In the individual regression analysis between MDZ concentration and awakening time, the mean MDZ concentration at awakening time was 16.8 ng/mL. The AUC of MDZ is the only significant factor associated with the awakening time.

## Introduction

Sedatives are frequently administered to mitigate undesirable physiological responses and alleviate discomfort during post-resuscitation care, including temperature control. Despite the numerous benefits associated with sedative administration, the effects of sedatives potentially confound neurological prognostication, thereby influencing decisions pertaining to the withdrawal of life-sustaining therapy in comatose patients after post-resuscitation care^1,2^.

Midazolam (MDZ) is frequently administered during post-resuscitation care because of its potential efficacy in managing suspected seizures^3^. However, MDZ has active metabolites with different pharmacological characteristics^4^; thus, it is important to identify these characteristics not only in the parent drug, but also in its active metabolites^5^. While the pharmacological activities of MDZ and its metabolites are well-known, drug metabolism is more variable in critically ill patients, especially in whom metabolic state is intentionally reduced to control body temperature control. It is strongly expected that drug metabolism and activity may be slower in these individuals compared to that in healthy volunteers^6–9^. These issues can render neuroprognostication more complex in post-resuscitation care, especially in determining the optimal and safe timing to avoid inappropriately pessimistic prognostications and therapeutic nihilism.

To understand the confounding effect of MDZ and propose a strategy to prevent neurologic death due to inappropriate pessimistic prognostication in patients with potential neurologic recovery, this study aimed to (1) investigate the distribution and elimination kinetics of MDZ and its known metabolites for the 24 h after discontinuation of MDZ continuous infusion, (2) determine the significant compound among MDZ and its metabolites associated with the sedative effect and evaluate the association of their pharmacokinetic (PK) characteristics with awakening time in patients with post-resuscitation care after cardiac arrest.

## Methods

### Study design and setting

A previous prospective single-center observational study conducted at our institution (IRB No. CNUH 2020-03-001-003) investigated the time-course relationship between cerebrospinal fluid and serum concentrations of MDZ in patients undergoing post-resuscitation care. The present study analyzed a subset of time serial data from this previous study regarding the concentration of MDZ and its metabolites after discontinuation of MDZ infusion (IRB No. CNUH 2023-11-019). This study was conducted according to the guidelines of the Declaration of Helsinki and approved by the IRB (or Ethics Committee) of our institution, CNUH. Written informed consent was obtained from all participants or their next of kin.

This prospective observational study included patients who received post-resuscitation care after an out-of-hospital cardiac arrest (OHCA) in May to September 2020 and May 2021 to February 2022. This study was interrupted from October 2020 to April 2021 due to the coronavirus disease 2019. This study included adult patients (aged > 18 years) who continuously received MDZ for sedation during post-resuscitation care, including temperature control. Among them, patients with a significant increase in serum creatinine of ≥ 25% or 0.5 mg/dL or reduction of estimated glomerular filtration by ≥ 25% of the baseline^10,11^, those receiving continuous renal replacement therapy, those who died before the complete discontinuation of midazolam infusion, those who did not provide informed consent, or those receiving extracorporeal membrane oxygenation were excluded from this study.

### Post-resuscitation care and midazolam administration

All patients received standard intensive care according to our institutional intensive care unit protocol based on the 2021 international guidelines for post-resuscitation care^2^. Patients who had a Glasgow Coma Scale (GCS) motor score of < 6 after return of spontaneous circulation underwent post-resuscitation arrest care. Temperature control was performed using a device for targeted temperature management of external cooling (Arctic Sun® 5000; BD, Franklin Lakes, NJ, USA). The targeted temperature was maintained for 24 h with rewarming to 37 ℃ at a rate of 0.25 ℃ per hour, and it was monitored using an esophageal or bladder temperature probe. MDZ (0.05–0.1 mg/kg intravenous bolus, followed by titrated intravenous continuous infusion at a rate of 0.1–1.0 mg/kg/h) was routinely administered for sedation and anti-epileptic effects. In addition to MDZ, a paralytic agent (rocuronium) and anti-epileptic drugs (lorazepam, levetiracetam, and/or valproate) were administered to control shivering caused by temperature control or to manage seizures, respectively. After temperature control, dose reduction (per 0.02 mg/kg) was performed to prevent iatrogenic withdrawal syndrome.

### Data collection and analysis

The characteristics of demographics and cardiac arrest and baseline information affecting PK of midazolam were collected in this study. Serum samples for analyzing the plasma concentration of MDZ and its metabolites were obtained immediately (baseline) and at 4, 8, 12, and 24 h after the discontinuation of MDZ infusion. All known MDZ metabolites were analyzed (see Supplementary Fig. S1 online). The sample preparation and methodology for concentration analysis are described in the Supplemental Methods, which are available online.

### Pharmacokinetic analysis

The PK parameters were obtained using a non-compartmental method with Phoenix WinNonlin (version 8.3.5; Certara, USA). The area under the time-plasma concentration curve (AUC) from time 0 to 24 h after the discontinuation of MDZ infusion (AUC_last_) was calculated using the trapezoidal linear interpolation rule. The AUC from time 0 to infinity (AUC_inf_) was calculated as AUC_last_ + C_last_/λ_z_, where C_last_ is the last measurable concentration and λ_z_ is the elimination rate constant. The mean residence time was calculated as the reciprocal of λ_z_. The estimated *t*_1/2_ was calculated from λ_z_ based on a previously reported equation^12^.

### Outcomes

The primary outcome of this study was the distribution and elimination of MDZ and its metabolites over 24 h following the discontinuation of MDZ infusion after targeted temperature management (33 or 36℃). The secondary outcome was the association between the PK parameters and awakening time in a subgroup of patients who experienced neurological recovery. Awakening time was defined based on two previous studies^13,14^: (1) if the patient opened their eyes spontaneously and (2) followed commands or visually tracked moving objects in response to a voice with total GCS score ≥ 9, or (3) showed a GCS motor score of 6.

### Statistical analysis

Categorical and continuous variables are presented as counts with percentiles and median values with interquartile ranges, respectively. The obtained PK parameters were described as means and standard deviations. Linear regression analysis was performed to demonstrate the association between the awakening time and PK parameters in the subgroup of awakened patients after post-resuscitation care. Adjustment with covariables showed a *p* value of < 0.1, and univariate linear regression was performed in multivariate linear regression. Backward selection was used to develop the final adjusted model. Statistical analyses were performed using SPSS version 26.0 for windows (IBM Corp., Armonk, NY, USA).

### Ethics approval and consent to participate

A previous prospective single-center observational study conducted at our institution (IRB No. CNUH 2020-03-001-003) investigated the time-course relationship between cerebrospinal fluid and serum concentrations of MDZ in patients undergoing post-resuscitation care. The present study analyzed a subset of time serial data from this previous study regarding the concentration of MDZ and its metabolites after discontinuation of MDZ infusion (IRB No. CNUH 2023-11-019). This study was conducted according to the guidelines of the Declaration of Helsinki and approved by the IRB (or Ethics Committee) of our institution, CNUH. Written informed consent was obtained from all participants or their next of kin.

## Results

### Baseline characteristics of total cohort

**Figure 1:**
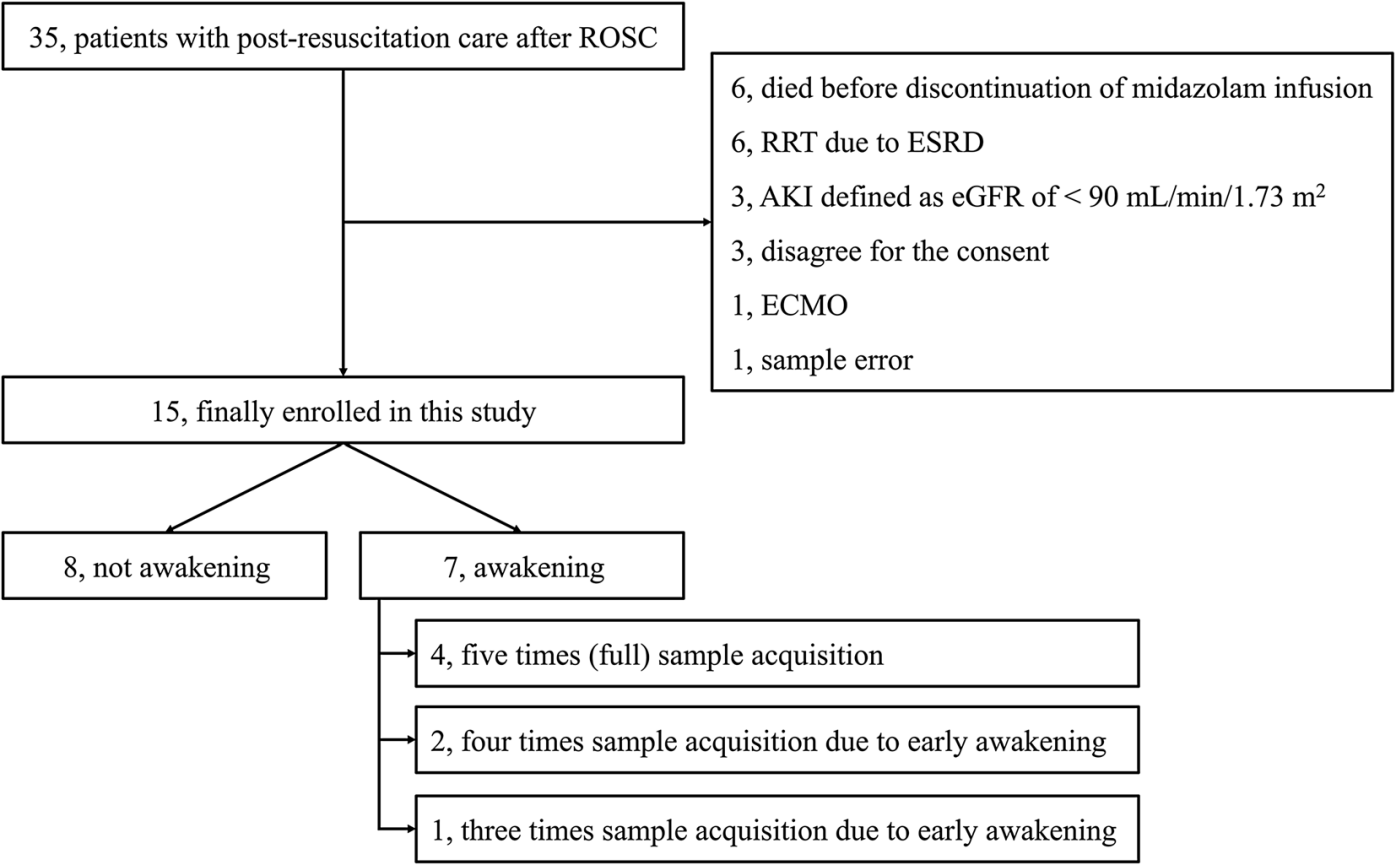
Flow diagram of included patients. *ROSC* return of spontaneous circulation; *AKI* acute kidney injury; *eGFR* estimated glomerular filtration rate; *RRT* renal replacement therapy; *ESRD* end-stage of renal disease; *ECMO* extracorporeal membrane oxygenation.

Of the 35 patients receiving post-resuscitation care after OHCA, 15 were finally enrolled in this study after the exclusion of 20 patients (Fig. 1). Out of the 15 enrolled patients, seven were previously included in a published study for the purpose of comparing serum and cerebrospinal fluid concentrations (Fig. S2)^15^. Of the finally enrolled 15 patients, 7 (46.7%) awakened after the discontinuation of MDZ administration (Fig. 1). Baseline demographics, cardiac arrest characteristics, and physiological status are shown in Table 1.

**Table 1:** Baseline demographics and characteristics.

Variables	Total study population, n = 15	Awakened patients n = 7
Age, years	59 (43–66)	60 (45–70)
Sex, male	9 (60.0)	5 (71.4)
Body surface area, m^2^	1.69 (1.52–1.79)	1.76 (1.52–1.88)
Charlson comorbidity index	2 (1–5)	
Cardiac arrest characteristics		
Witnessed	9 (60.0)	6 (85.7)
Bystander CPR	9 (60.0)	4 (57.1)
Shockable rhythm	7 (46.7)	6 (85.7)
Cardiac etiology	7 (46.7)	6 (85.7)
No-flow time, min	2 (0–16)	1 (0–3)
Low-flow time, min	18 (9–25)	9 (6–25)
Total dose of administered sedatives		
Midazolam, mg/kg/h	0.15 (0.10–0.18)	0.15 (0.11–0.17)
Levetiracetam, mg/kg/d	23.3 (17.2–63.5)	18.8 (15.5–72.7)
Lorazepam, mg	5.0 (4.0–6.5)	4 (4–4)
Body temperature at sample acquisition, °C		
Immediately after discontinuation of midazolam infusion (0 h)	36.8 (36.5–37.3)	37.0 (36.0–38.0)
At 4 h	36.8 (36.1–37.4)	36.8 (36.0–38.0)
At 8 h	36.7 (36.6–37.2)	37.1 (36.6–37.7)
At 12 h	36.8 (36.7–37.2)	37.1 (36.8–37.4)
At 24 h	36.8 (36.6–37.3)	36.7 (36.2–36.9)
^a^TWA until discontinuation of midazolam infusion		
Hepatic function		
Total bilirubin, mg/dL	0.5 (0.4–0.8)	0.6 (0.5–1.1)
Alkaline phosphatase, U/L	58.4 (38.4–69.3)	50.5 (40.9–65.2)
Alanine transaminase, U/L	35.6 (25.4–48.7)	35.6 (23.0–67.6)
Aspartate aminotransferase, U/L	45.1 (32.4–63.0)	46.1 (28.5–61.2)
Renal function		
Creatinine, mg/dL	0.60 (0.47–0.68)	0.62 (0.55–0.80)
eGFR, mL/min/1.73 m^2^	118.3 (111.2–131.3)	113.2 (105.6–128.3)

Data are presented as n (%) or medians (interquartile ranges).

^a^To consider the numerical exposure levels of hepatic and renal dysfunction with the amount of time spent on it, the TWA was calculated until the discontinuation of midazolam infusion from admission. We multiplied the length of time the patient spent at a specific marker level by that marker value, added all these values, and divided the result by the total observation time. Thus, the equation is$TWA=\frac{{\sum }_{k=1}^{n}{value}_{k}\times {time}_{k}}{\sum_{k=1}^{n}{time}_{k}}$.

CPR, cardiopulmonary resuscitation; TWA, time-weighted average; eGFR, estimated glomerular filtration rate.

### Distribution of midazolam and its metabolites in plasma

**Figure 2:**
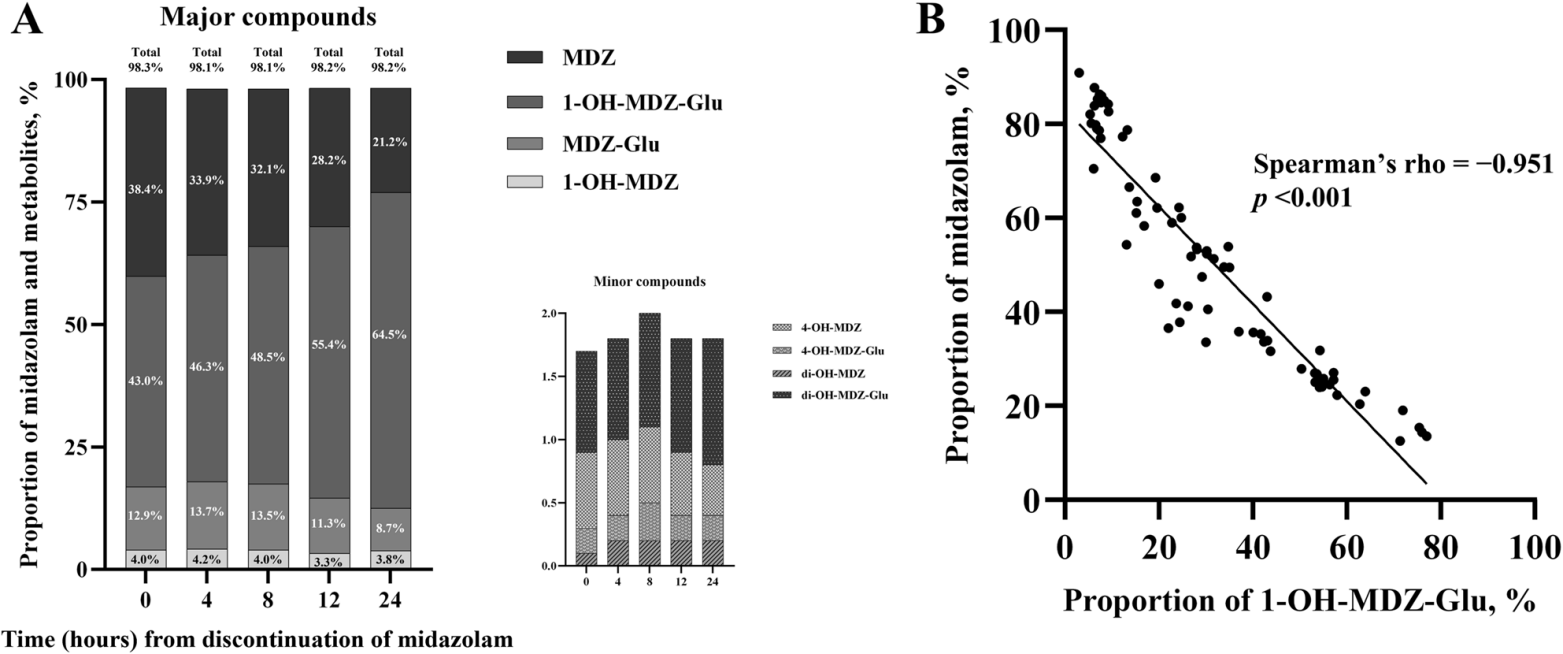
Distribution of midazolam and its major and minor metabolites following discontinuation of midazolam infusion. MDZ, midazolam; 1-OH-MDZ-Glu, 1-hydroxymidazolam glucuronide; MDZ-Glu; midazolam glucuronide; 1-OH-MDZ, 1-hydroxymidazolam; di-OH-MDZ-Glu, di-hydroxymidazolam glucuronide; 4-OH-MDZ, 4-hydroxymidazolam; 4-OH-MDZ-Glu, 4-hydroxymidazolam glucuronide; di-OH-MDZ, di-hydroxymidazol.

The distribution of each compound was assessed by calculating its relative concentration as a ratio to the total sum of relative concentrations at each sampling time. Figure 2 illustrates the time-dependent changes in the distribution of MDZ and its metabolites following MDZ administration discontinuation. Major compounds; MDZ, 1-hydroxymidazolam-glucuronide (1-OH-MDZ-Glu), midazolam glucuronide (MDZ-Glu), and 1-hydroxymidazolam (1-OH-MDZ); constituted the predominant proportion, accounting for approximately 98% of the total, whereas minor metabolites constituted less than 2% (Fig. 2). The proportion of MDZ and 1-OH-MDZ-Glu in the total concentration showed a significant negative correlation (Spearman’s rho =  − 0.95, *p* < 0.001, Fig. 2).

### Elimination profile of the midazolam and its metabolites concentrations

**Figure 3:**
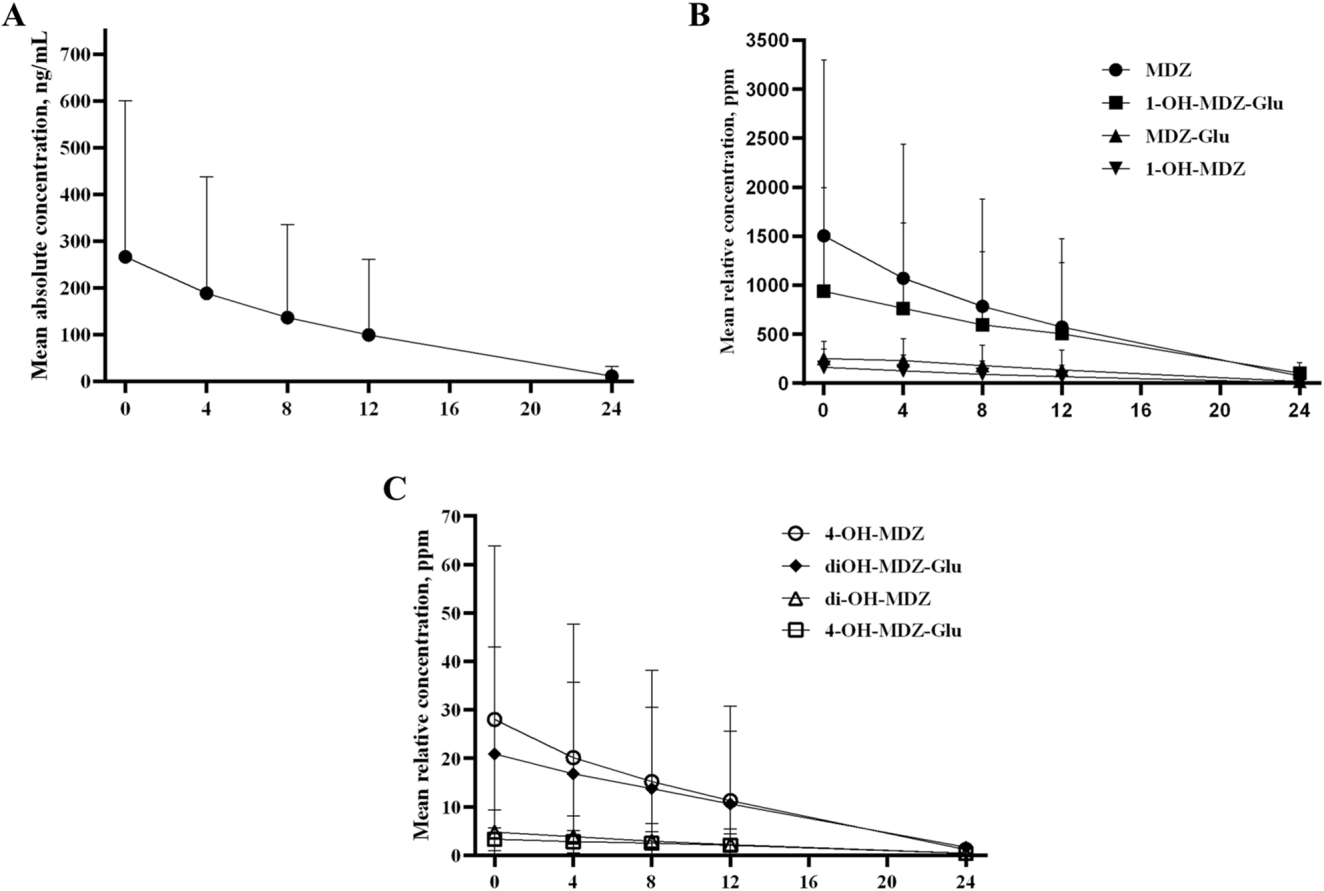
Elimination profile of all compounds after discontinuation of the midazolam infusion (hours, x-axis) in total cohort. The changes in the absolute concentration (ng/mL) of midazolam (**A**) and the relative concentration (ppm) of the major (**B**) and minor (**C**) compounds are shown. Data are presented as the mean value and standard deviation (upper error bars; lower error bars omitted for clarity). MDZ, midazolam; 1-OH-MDZ-Glu, 1-hydroxymidazolam glucuronide; MDZ-Glu; midazolam glucuronide; 1-OH-MDZ, 1-hydroxymidazolam; di-OH-MDZ-Glu, di-hydroxymidazolam glucuronide; 4-OH-MDZ, 4-hydroxymidazolam; 4-OH-MDZ-Glu, 4-hydroxymidazolam glucuronide; di-OH-MDZ, di-hydroxymidazol.

Figure 3 illustrates the time-dependent elimination profile of the concentrations of the MDZ and its metabolites. Despite variations in the total administered dose of MDZ among individuals, the elimination patterns of MDZ and its metabolites following the discontinuation of continuous infusion demonstrated a first-order elimination profile in all patients (Fig. 3). The elimination rate showed over 50% inter-individual variability, likely attributable to differences in hepatic metabolic rates among the critically ill patients (Fig. 3).

### Pharmacokinetic parameters and their association with awakening time in the awakening group

**Figure 4:**
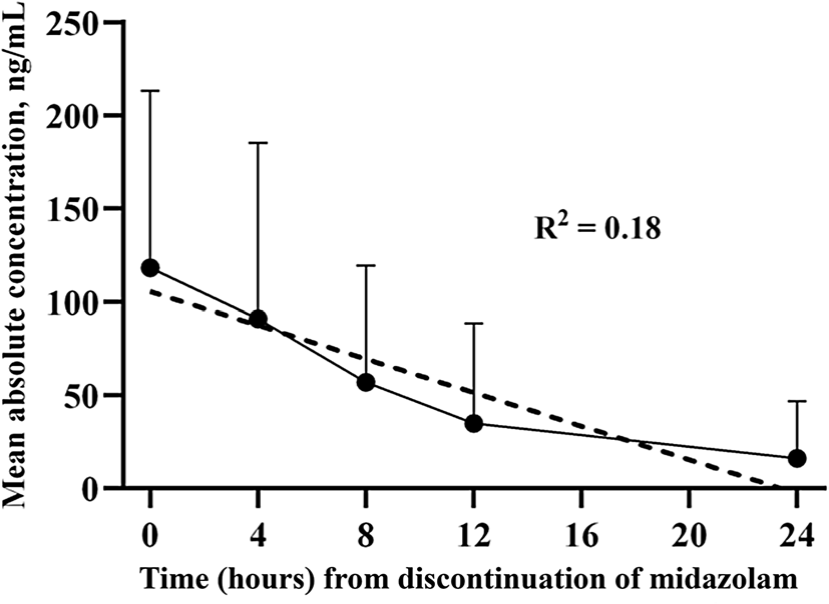
Simple linear regression analysis between the absolute concentration of midazolam and awakening time in the subgroup of awakened patients after post-resuscitation care (n = 7). Data are presented as the arithmetic mean values (black circles) and standard deviations (upper error bars; lower error bars omitted for clarity). The regression line (grid line) is followed the regression equation: Y =  − 4.506 × X + 105.5. Since the awakening time in the subgroup of this study was 16.6 h after the discontinuation of the midazolam infusion, the estimated concentration of the midazolam at that time is 30.7 ng/mL.

We investigated the PK parameters for all compounds in each individual. Among them, MDZ showed an estimated* t*_1/2_ of 6.2 h (Table 2). Table 3 shows the linear regression analysis between awakening time and the estimated AUC_last_ and AUC_inf_ for all compounds in seven patients with neurological recovery post-resuscitation care. Although MDZ and a few metabolites were initially associated with awakening time, multivariate analysis revealed that only MDZ itself was significantly associated with awakening time, specifically in terms of the PK parameter AUC_last_ (β = 0.001, 95% confidence interval [CI] = 0.000–0.002, adj. R^2^ = 0.59, *p* = 0.03; Table 3) and AUC_inf_ (β = 0.001, 95% CI = 0.001–0.001, adj. R^2^ = 0.96, *p* < 0.001; Table 3). When comparing the association between independent PK parameters and awakening time, AUC_inf_ exhibited a higher explanatory accuracy (adjusted R^2^ value, 0.96 vs. 0.59, Table 3) compared to that of AUC_last_. Figure 4 shows that simple linear regression using the MDZ absolute concentration and awakening time in seven awakened patients. Following this equation, Table 4 shows that the MDZ concentration at the time of awakening was predictable in four out of seven patients. Among these four patients, the mean of the estimated MDZ concentration was 16.8 ng/mL.

**Table 2:** Pharmacokinetic parameters of midazolam and its metabolites.

	PK parameters				
	AUC_last_ (^a^unit)	AUC_inf_ (^a^unit)	*t*_1/2_ (h)	MRT_last_ (h)	MRT_inf_ (h)
Parent drug					
MDZ	1772.0 ± 2742.3	2106.7 ± 3053.8	6.2 ± 3.9	4.9 ± 1.6	8.2 ± 5.2
Major metabolites					
1-OH-MDZ-Glu	9193.0 ± 13,958.4	14,027.8 ± 15,486.2	15.4 ± 19.9	6.8 ± 2.4	21.8 ± 26.8
MDZ-Glu	2175.6 ± 2212.3	3158.5 ± 2887.2	11.4 ± 17.0	5.8 ± 1.9	16.2 ± 24.1
1-OH-MDZ	1117.2 ± 1643.5	1419.1 ± 1845.5	8.1 ± 4.9	6.0 ± 2.3	10.5 ± 5.2
Minor metabolites					
4-OH-MDZ	172.5 ± 268.0	204.7 ± 294.8	7.3 ± 3.8	5.6 ± 1.5	10.0 ± 4.8
4-OH-MDZ-Glu	29.8 ± 24.4	33.3 ± 34.3	5.4 ± 0.7	6.4 ± 2.2	7.9 ± 1.4
Di-OH-MDZ	38.2 ± 49.8	56.2 ± 52.1	14.5 ± 13.7	6.4 ± 2.5	21.0 ± 19.8
Di-OH-MDZ-Glu	187.4 ± 272.0	200.2 ± 307.1	14.3 ± 9.6	6.4 ± 2.1	19.2 ± 13.0

Data are presented as arithmetic means ± standard deviations.

^a^The units of AUC values were different between MDZ (h*ng/mL) and metabolites (h*ppm), since those were analyzed as absolute and relative concentration, respectively.

PK, pharmacokinetic; SD, standard deviation; AUC, area under the curve; t_1/2_, elimination half-life; MRT, mean residence time; MDZ, midazolam; 1-OH-MDZ-Glu, 1-hydroxymidazolam glucuronide; MDZ-Glu, midazolam glucuronide; 1-OH-MDZ, 1-hydroxymidazolam; 4-OH-MDZ, 4-hydroxymidazolam; 4-OH-MDZ-Glu, 4-hydroxymidazolam glucuronide; di-OH-MDZ, dihydroxymidazolam; di-OH-MDZ-Glu, dihydroxymidazolam glucuron.

**Table 3:** Multivariate linear regression analysis of pharmacokinetic parameters for awakening time.

	AUC_last_ (^a^unit)				AUC_inf_ (^a^unit)			
	Univariable		^b^Multivariable Adj. R^2^ = 0.59		Univariable		^b^Multivariable Adjusted R^2^ = 0.96	
	β (95% CI)	*p*	β (95% CI)	*p*	β (95% CI)	*p*	β (95% CI)	*p*
Parent drug								
MDZ	0.001 (0.000–0.002)	0.03	0.001 (0.000–0.002)	0.03	0.001 (0.001–0.001)	< 0.001	0.001 (0.001–0.001)	< 0.001
Major metabolites								
1-OH-MDZ-Glu	0.000 (–0.004–0.003)	0.80			0.002 (0.000–0.003)	0.03	NS	0.79
MDZ-Glu	0.004 (–0.013–0.022)	0.55						
1-OH-MDZ	0.029 (–0.001–0.059)	0.06	NS	0.64	0.015 (0.003–0.027)	0.02	NS	0.30
Minor metabolites								
4-OH-MDZ	0.125 (0.007–0.243)	0.04	NS	0.97	0.046 (–0.010–0.102)	0.09	NS	0.96
4-OH-MDZ-Glu	–0.262 (–1.024–0.501)	0.42			0.019 (–0.018–0.057)	0.24		
di-OH-MDZ	0.238 (–1.349–1.825)	0.72			0.056 (–0.038–0.149)	0.19		
di-OH-MDZ-Glu	–0.047 (–0.237–0.143)	0.55			0.021 (–0.118–0.160)	0.71		

Regression coefficients (β) with 95% confidence intervals (95% CIs) and *p*–values are shown for univariate and multivariate linear regression models of pharmacokinetic parameters with awakening time in patients with neurological recovery.

^a^The units of AUC values were different between MDZ (h*ng/mL) and metabolites (h*ppm), since those were analyzed as absolute and relative concentration, respectively.

^b^Covariables with a *p* value < 0.1 in univariate analysis were included in the multivariate analysis, and adjusted R^2^ values were reported. Units of concentration in all compounds and awakening time were ppb and hour, respectively.

AUC_last_, area under the plasma concentration–time curve from time 0 to the last measurable time point; AUC_inf_, area under the plasma concentration–time curve from time 0 to infinity; NS, non-specific; MDZ, midazolam; 1-OH-MDZ-Glu, 1-hydroxymidazolam glucuronide; MDZ-Glu, midazolam glucuronide; 1-OH-MDZ, 1-hydroxymidazolam; 4-OH-MDZ, 4-hydroxymidazolam; 4-OH-MDZ-Glu, 4-hydroxymidazolam glucuronide; di-OH-MDZ, dihydroxymidazolam; di-OH-MDZ-Glu, dihydroxymidazolam glucuronid.

**Table 4:** Estimated absolute concentration of midazolam at the time of patient awakening using simple linear regression analysis.

Subject	Awaken time after discontinuation of midazolam intravenous infusion (h)	Estimated midazolam concentration at awaken time (ng/mL)
#1	9.6	32.3
#2	10.4	20.8
#3	9.6	2.2
#4	7.1	11.9

## Discussion

MDZ and three metabolites (1-OH-MDZ-Glu, MDZ-Glu, and 1-OH-MDZ) accounted for approximately 98% of the total concentration. In addition, 1-OH-MDZ-Glu had the largest proportion at all time points and an almost perfect negative correlation with MDZ in comparison to the proportion of the total concentration. The hydroxylation of MDZ in the liver is the first step in its metabolism. Consequently, two metabolites are formed, 1-OH-MDZ and 4-hydroxymidazolam (4-OH-MDZ). Following hydroxylation, the glucuronide conjugates are eliminated via renal secretion. A previous study reported that 1-OH-MDZ-Glu was generally associated with sedation effect in high concentration due to its pharmacologically very low potency of < 6%^16,17^. Given the previous reports and our finding of a non-significant association between 1-OH-MDZ-Glu and awakening time, we suggest that 1-OH-MDZ-Glu is just the final product of MDZ metabolism and is not associated with sedative effects. Therefore, it may not be a factor in determining the optimal or safe time for neuroprognostication in post-resuscitation care, despite having the largest proportion of relative concentration among all compounds.

We investigated the association between the PK parameters of all compounds and awakening time in the subgroup. The AUC value in PK analysis provides valuable information about the overall drug concentration profile and is a key parameter used to assess the extent of drug exposure over a specific period^18^. The AUCs in few metabolites, namely, 1-OH-MDZ, 4-OH-MDZ, and 1-OH-MDZ-Glu showed a significant association with awakening time in this study, whereas their association was not significant in the multivariable analysis. In line with this study, previous studies revealed that 4-OH-MDZ (0–2%) and 1-OH-MDZ-Glu (6–10%) have low potency for sedation^16^. In addition, the proportion of 1-OH-MDZ in total concentration was significantly less than MDZ itself (3.3–4.2% vs. 21.2–38.4%) despite its relatively higher potency for sedation effect (60–63%)^19,20^. Based on previous studies and our findings, it appears that the concentration of MDZ itself is the primary factor associated with awakening time in patients with potential neurologic recovery following post-resuscitation care, rather than its metabolites.

A 0.001% increase in MDZ AUC was associated with a 1% increase in awakening time. The current guideline for the prognostication in patients with post-resuscitation care recommends waiting 12 h after discontinuation of sedative infusions before prognostication^1,2^. However, none of the pharmacologic concentrations in the absence of confounding effects that might lead to the inappropriate withdrawal of life-sustaining therapy have been covered yet. Although we found that AUC_inf_ exhibited the strongest association with awakening time with a high accuracy level (adj. R^2^ = 0.96), we recommend using AUC_last_ as a predictive factor for awakening time. This is because AUC_inf_ may not a reliable indicator for predicting pessimistic prognosis since it involves extrapolation during the calculation process, which requires stronger empirical evidence for real-world clinical application. Considering our results and a previous study for the individual PK characteristics even in the same confounder^21,22^, we suggest that it is essential to estimate the concentration of the major confounder for determining the optimal and safe timing to avoid inappropriate pessimistic prognostications and therapeutic nihilism, rather than solely relying on the empirically known time.

In patients receiving post-resuscitation care after cardiac arrest, the sedative drug concentration is not the sole factor affecting the awakening time given the involvement of other factors, including cerebral dysmetabolism, microcirculatory dysfunction, and impaired autoregulation^23–25^. Therefore, our findings suggest that elucidating the elimination kinetics of MDZ can at least ensure that the confounding effect of MDZ is not ignored prior to reaching a certain time (or concentration). Furthermore, we suggest that withdrawal of life-sustaining treatment should not be determined solely based on the MDZ plasma concentration.

This study has some limitations. Most notably, this study had a small sample size. Since this study was interrupted and terminated due to coronavirus disease 2019 and limited fund, only the data from seven patients who had neurological recovery could be used to analyze the association between PK parameters and awakening time. Therefore, our study may have been underpowered. Although the MDZ elimination profile and the estimated t_1/2_ were similar to those observed in previous studies^26,27^, future studies with larger samples are required to generalize our results. Other covariables associated with PK, such as hepatic enzyme function or drug interactions, were not included in this analysis, which created bias in confirming the PK parameters of MDZ and its metabolites. However, we found that all patients included in this study had normal liver and kidney functions through blood chemistry analysis 24 h after the discontinuation of MDZ infusion. Although the study protocol warranted deep sedation (Richmond Agitation Sedation Scale − 4 to − 5), data on level of sedation was not collected. The association of the anti-epileptic drugs, such as levetiracetam or lorazepam, was not considered in this study. However, lorazepam was administered in the emergency department to manage clinically observed seizures and was not repeated in intensive care unit; moreover, levetiracetam was routinely administered to patients who had seizures observed during the electroencephalography performed within 24 h of cardiac arrest. It is noteworthy that a prior study, seizures were not associated with the doses of propofol or midazolam, indicating the adequate treatment effect of antiepileptic drugs without a need for increased sedation^28^. Nonetheless, the issue of administered anti-epileptic drugs can lead to a significant bias to this study. Six patients who were already enrolled and undergoing concentration measurements, were excluded due to the death during the study period. Moreover, three patients had neurological recovery after post-resuscitation care and were awakened within 24 h after the discontinuation of MDZ infusion; thus, their samples were not collected at all five time points (baseline, 4, 8, 12, and 24 h after the discontinuation of MDZ infusion). This is a significant issue leading to a selection bias.

## Conclusion

After discontinuation of MDZ infusion for prognostication after post-resuscitation care, four major compounds (MDZ, 1-OH-MDZ-Glu, MDZ-Glu, and 1-OH-MDZ) have a dominant proportion exceeding 98%. Their elimination kinetics follow a first-order elimination profile despite the variations in the total administered dose of MDZ among individuals. Among the PK parameters of MDZ and its metabolites, only the AUC of MDZ itself is significantly associated with the awakening time. When making decisions regarding the withdrawal of life-sustaining therapy for patients receiving post-resuscitation care after return of spontaneous circulation, it is important to consider the confounding effect of MDZ. Further well-designed prospective studies are warranted to improve the generalizability of our results.

### Supplementary Information

Supplementary Information.

## Data Availability

The data presented here is available on request from the corresponding author. The data are not publicly available because of ethical concerns.
