# Supplementary material for: Distribution and elimination kinetics of midazolam and metabolites after post-resuscitation care: a prospective observational study
Source: Sci Rep. 2024 Feb 25;14:4574. doi: 10.1038/s41598-024-54968-z (PMC10894853; doi:10.1038/s41598-024-54968-z)
Supplement: Supplementary file 1 — Supplementary Information. [file 41598_2024_54968_MOESM1_ESM.docx]

**Supplemental materials**

**Distribution and elimination kinetics of midazolam and metabolites after post-resuscitation care: A prospective observational study**

**Figure S1.** Metabolites and their metabolic pathway of midazolam

**Figure S2.** Flow diagram of a previous study and the present study

**eMethod 1.** Sample preparation for analysis

**eMethod 2**. Liquid chromatography with tandem mass spectrometry (LC-MS/MS) analysis

**Figure S3**. MS/MS spectra of midazolam and its metabolites

**Table S1**. MRM transition and MS parameters for monitoring of midazolam and its metabolites

**Figure S4.** chromatogram of midazolam and each metabolite.

**Table S2.** Linearity, range, and coefficient of determination of midazolam

**Table S3.** Recovery of midazolam in human serum and CSF during sample preparation

**Figure S5.** Calibration curve of midazolam with range of (A) 0.1 – 5.0 ng/mL and (B) 5.0 – 100.0 ng/mL


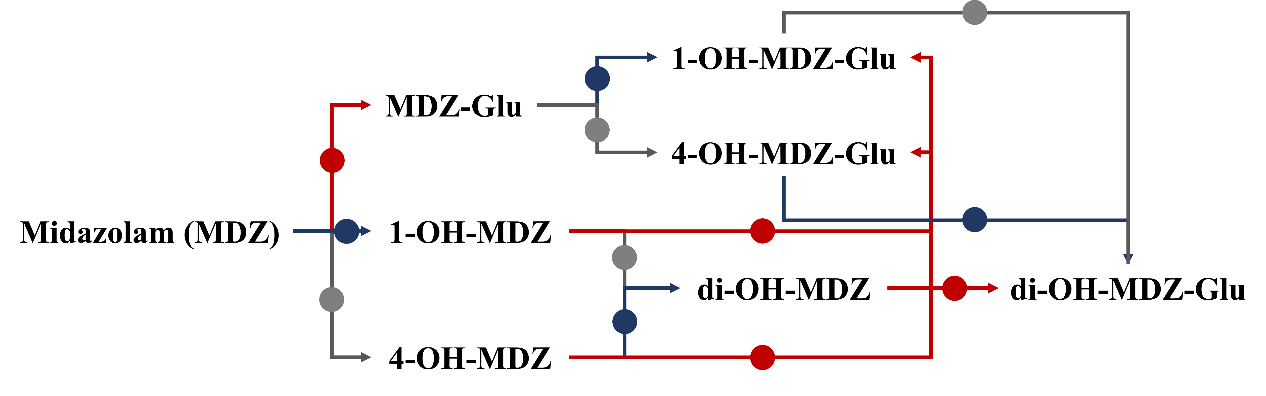


**Figure S1.** Midazolam metabolites and their metabolic pathways. The metabolism of midazolam involves two enzymatic steps: the cytochrome P450 3A4/3A5 enzymes that hydroxylate midazolam to form 1- (blue line) or 4- (gray line) hydroxymidazolam and the UGT 2B4/2B7 and 1A4 enzymes that conjugate hydroxymidazolam with glucuronic acid to form hydroxymidazolam glucuronide (red line).

***Abbreviation***: MDZ, midazolam; 1-OH-MDZ-Glu, 1-hydroxymidazolam glucuronide; MDZ-Glu; midazolam glucuronide; 1-OH-MDZ, 1-hydroxymidazolam; di-OH-MDZ-Glu, di-hydroxymidazolam glucuronide; 4-OH-MDZ, 4-hydroxymidazolam; 4-OH-MDZ-Glu, 4-hydroxymidazolam glucuronide; di-OH-MDZ, di-hydroxymidazolam

**Figure S2.** Flow diagram of a previous study and this study

**
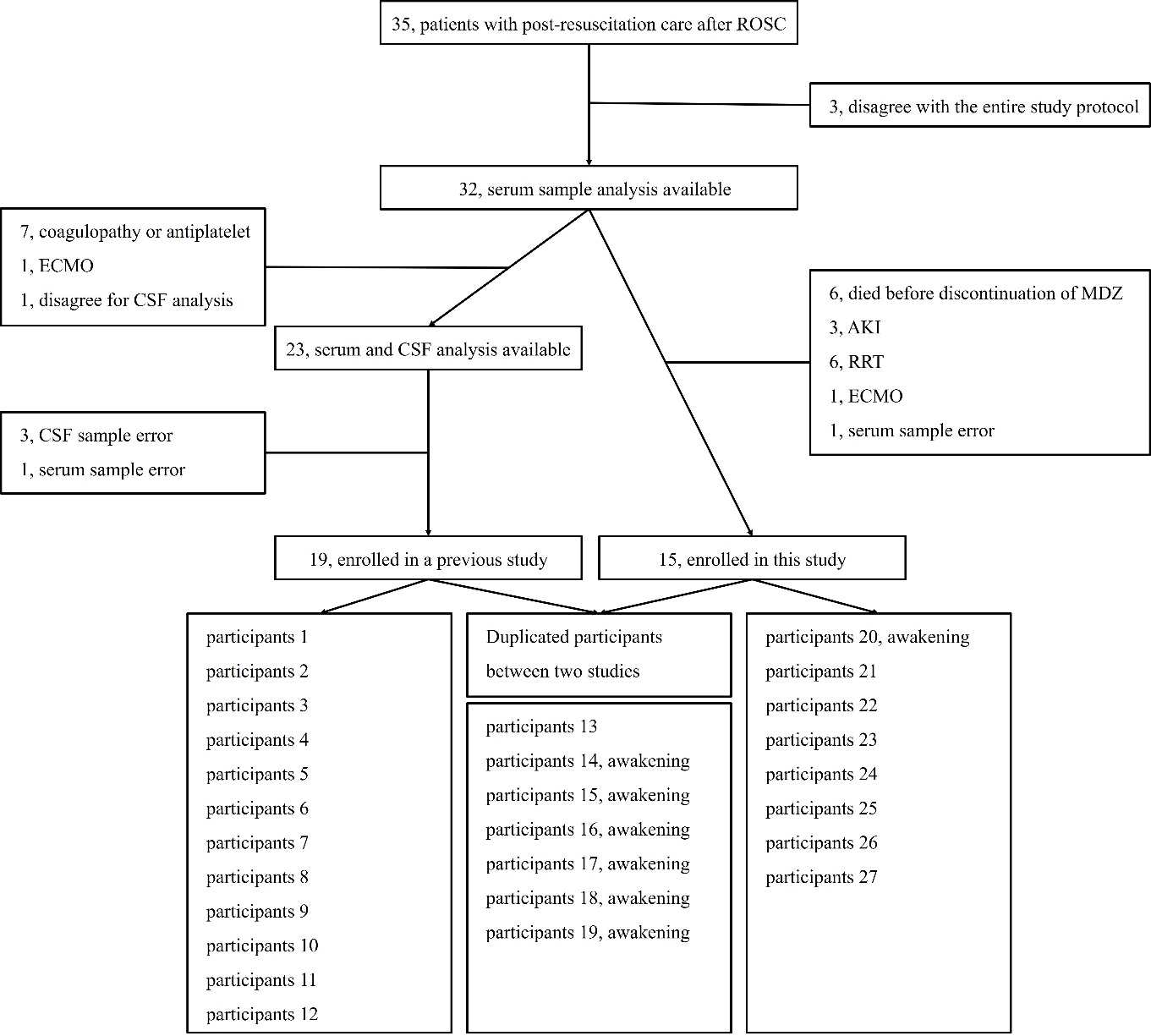
**

**eMethod 1.** Sample preparation for analysis

All samples were immediately frozen and stored at –70 ℃ after centrifugation with 3,000 rpm for 10 min and then sent to the Nutrition Food Microbiology Laboratory at Chungnam National University (http://nfml.kr/) to determine the concentration of midazolam and its metabolites using liquid chromatography with tandem mass spectrometry analysis.

The sample preparation method was performed as previously described [1, 2] with some modifications. A 20 µL of sample was mixed with 180 µL of 80% methanol, followed by protein precipitation at 4 °C for 30 min. Subsequently, the supernatant was harvested and added to 1800 µL of distilled water. The pre-treated samples were loaded onto an Oasis HLB Vac RC cartridge (30 mg, 20 cc, Waters) that had been preconditioned with 6 mL of methanol and 6 mL of distilled water. The cartridge was washed with 6 mL distilled water and sequentially eluted with methanol (4 mL). The eluted samples were dried using an evaporator and resuspended with 0.2 mL of methanol for analysis.

**eMethod 2.** Liquid chromatography with tandem mass spectrometry (LC-MS/MS) analysis

Midazolam (MDZ) was quantitatively analysed by a multiple reaction monitoring (MRM) method using triple quadrupole mass spectrometry coupled with liquid chromatography. Chromatographic separation was performed on an Agilent 1290 UPLC system (Agilent Technologies, USA) equipped with a binary pump, online degasser, autosampler, and a thermostatically controlled column compartment. Chromatographic separation was performed using an Ascentis Express C18 HPLC column (10 cm × 2.1 mm, 2.7 µm, Merck, Germany) for the elution of midazolam. The mobile phase consisted of solvents A (0.1% formic acid in distilled water) and B (0.1% formic acid in acetonitrile). The mobile phase was delivered at a flow rate of 300 μL/min, and the injection volume was 3 μL. The gradient was as follows: at 0.0–5.0 min 5% B, at 5.0–10.0 min increase to 40% B, at 10.0–15.0 min linear increase to 97% B, at 15.0–15.5 min increase 100% B, at 15.5–20.5 min wash with 100% B, and at 20.5–21.0 min drop to 5% B, and remained constant for 14 min before returning to the initial conditions and re-equilibrating. In conjunction with the LC unit, an Agilent 6470 LC-MS/MS triple quadrupole (Agilent Technology, USA) was used for mass spectrometric analysis. The nebulizer, dry gas flow, and dry gas temperature were set at 45 psi, 9.0 L/min, and 350 °C, respectively. The sheath gas heater and flow were set at 350 °C and 11 L/min, respectively. The positive ESI-MRM was employed using precursor to product ion transitions of m/z 326.2→291.2 and m/z 326.2→249.1 for midazolam quantitation.

In this study, midazolam, 1-hydroxymidazolam, and 4 hydroxymidazolam were obtained from Sigma Aldrich (Merck To determine other midazolam metabolites, a manual selection process was employed due to lack of available standards. In addition to the hydroxylation, a glucuronidation of MDZ, OH-MDZ, and diOH-MDZ were considered. The theoretical precursor ions of each metabolite were extracted through extract ion chromatogram after scanning the actual sample. The expected peaks of the hydroxy- and glucuronidated metabolites of MDZ must have retention time earlier than MDZ itself. Then, the MS/MS spectra of each MDZ metabolite was studied to confirm the structure. The fragments of 203.1 [X_2_] and 233.9 [Y_2_] were the signature for 1-OH-MDZ and 4-OH-MDZ, respectively (Fig. S2 A, B, C). The fragments [X] and [Y] of 1-OH-MDZ, 4-OH-MDZ, and di-OH-MDZ closely matched those from previous studies [3, 4].

In the mass spectra of di-hydroxymidazolam, there were fragments of dehydrate-forms [M-H_2_O]^+^, [M-H_2_O]+, and [Y_2_] (Fig. S2 D). To identify the glucuronidated MDZ, fragments of the MDZ core structure (m/z 326.2, 291.1) were indicators for the corresponding precursor. Similarly, the glucuronidated of OH-MDZ or di-OH MDZ spectra also contained fragments of aglycones (m/z 342.1 or 358.1) and their dehydrate-form [M-Glu-H_2_O]^+^ such as m/z 324.1 and 340.1 respectively. The position of glucuronide was identified by signature fragments (Fig. S2 E, F, G, H).

After the precursor and product ions were determined, the MRM transition for each metabolite was selected. For the quantification of midazolam and its metabolites, MRM was transitioned in the positive ion mode (Table S1). The MRM chromatogram of midazolam and its metabolites is shown in Figure S1.

The analytical method was performed as previously described [1, 2], with some modifications. The recovery of midazolam extracted from both serum and cerebrospinal fluid (CSF) was performed using the standard addition method as follows:

$$Recovery \left( \% \right)=\frac{Cspiked sample-Cunspiked sample}{Cadded standard}\times100$$

The average recovery percentages for midazolam in human serum and CSF were within the 95%–105% range, exhibiting a coefficient of variation (CV) ≤5% (refer to Table S3). The obtained data was consistent with previous findings, confirming the efficacy of the established extraction and purification approach for midazolam in human serum and CSF. Similarly, the recovery of 1- and 4-hydroxymidazolam were evaluated using the same method. The recovery of 1- and 4-hydroxymidazolam was 90.5% ± 3.5% and 95.5% ± 5.2 %, respectively.

To measure a wide range of midazolam concentration, two linear regression equations which ranged from 0.1-5.0 and 5.0-100.0 ng/mL were generated (Table S2). The low limit of detection and quantitation were 30 and 100 pg/mL. The coefficient of determination of the calibration curves was 0.9996 (Fig. S4).

Figure S3. MS/MS spectra of midazolam and its metabolites; MDZ (A); 1-hydroxyMDZ (B); 4-hydroxyMDZ(C); dihydroxyMDZ (D); MDZ-Glucuronide (E); 1-hydroxyMDZ-Glucuronide (F); 4-hydroxyMDZ-Glucuronide (G); dihydroxyMDZ-Glucuronide (H)


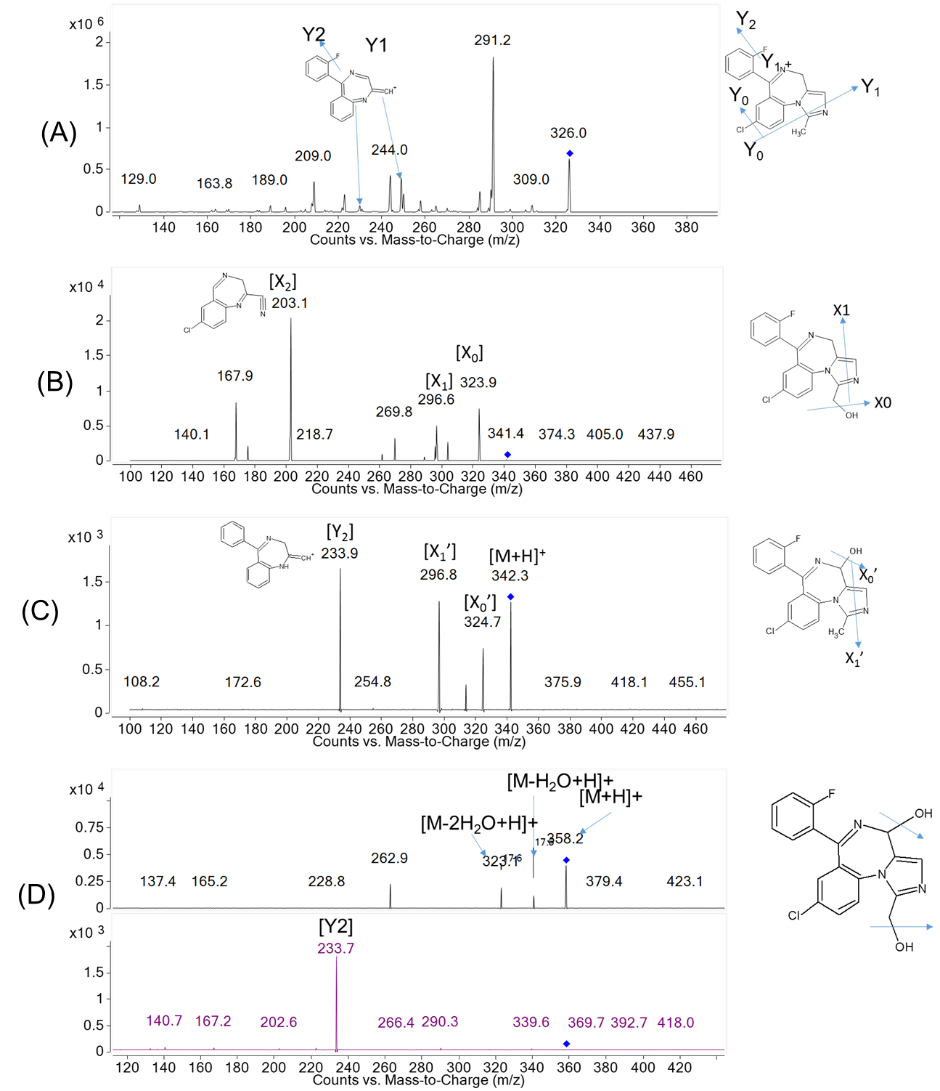

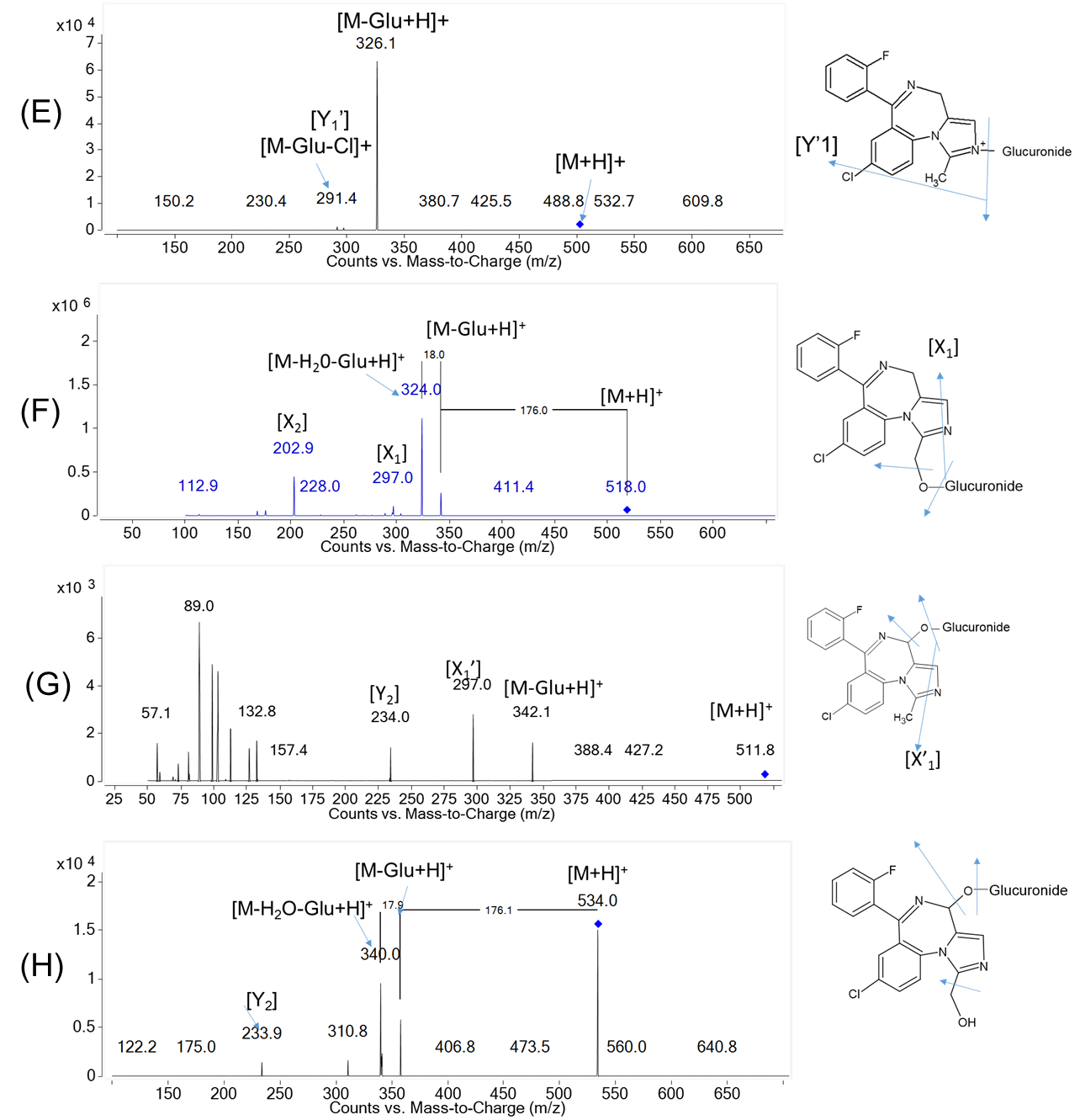


**Table S1.** MRM transition and MS parameters for monitoring of the midazolam and its metabolites

|  | MRM transition (m/z)  Precursor ion → product ion | | Fragment (V) | Collision Energy (V) | Cell Accelerator(V) |
| --- | --- | --- | --- | --- | --- |
| MDZ | 326.2 | 291.2 | 130 | 30 | 4 |
|  |  | 249.1 | 130 | 25 | 4 |
|  |  | 233.9 | 130 | 25 | 4 |
| 1-OH MDZ | 342.1 | 202.9 | 100 | 20 | 5 |
|  |  | 324.0 | 100 | 20 | 4 |
| 4-OH MDZ | 342.1 | 234.0 | 130 | 25 | 5 |
|  |  | 297.2 | 100 | 20 | 5 |
| di-OH-MDZ | 358.2 | 233.6 | 100 | 20 | 4 |
|  |  | 340.1 | 100 | 18 | 4 |
| MDZ-Glu | 502.2 | 291.3 | 90 | 40 | 4 |
|  |  | 326.1 | 90 | 27 | 4 |
| 1-OH-MDZ-Glu | 518.1 | 202.6 | 130 | 40 | 5 |
|  |  | 324.1 | 130 | 25 | 5 |
| 4-OH-MDZ-Glu | 518.1 | 233.9 | 130 | 38 | 5 |
|  |  | 325.0 | 130 | 40 | 5 |
| di-OH-MDZ-Glu | 534.2 | 340.0 | 130 | 25 | 4 |
|  |  | 358.1 | 130 | 30 | 4 |


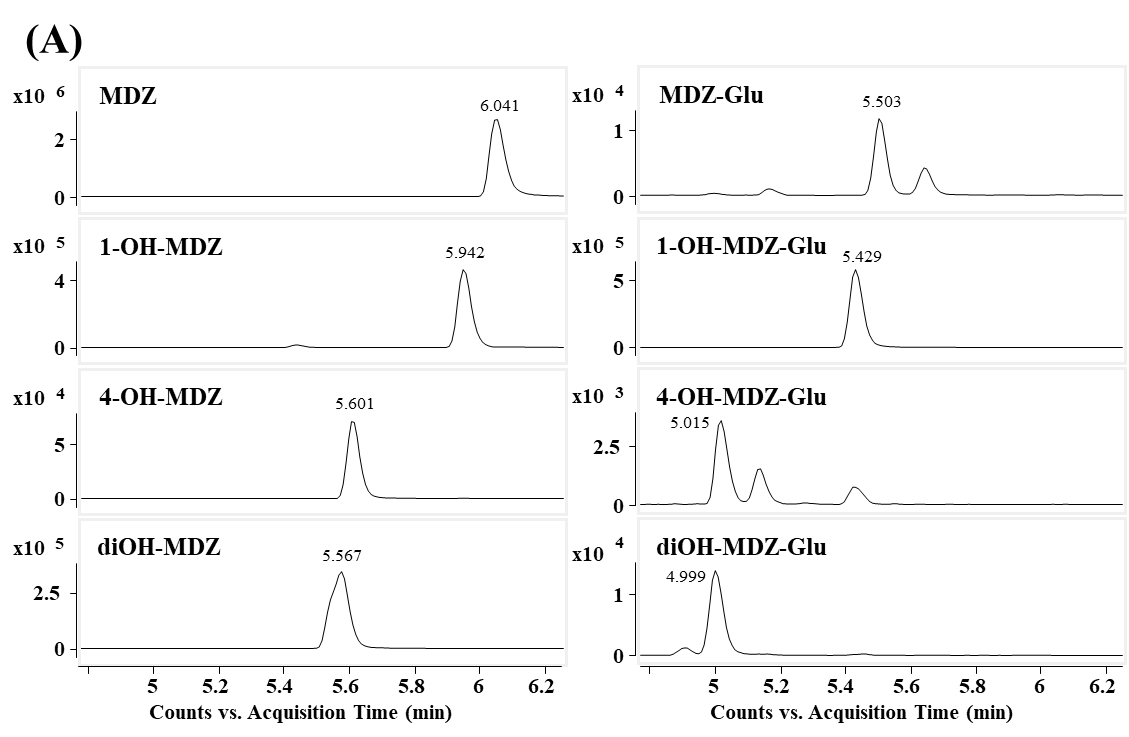
**
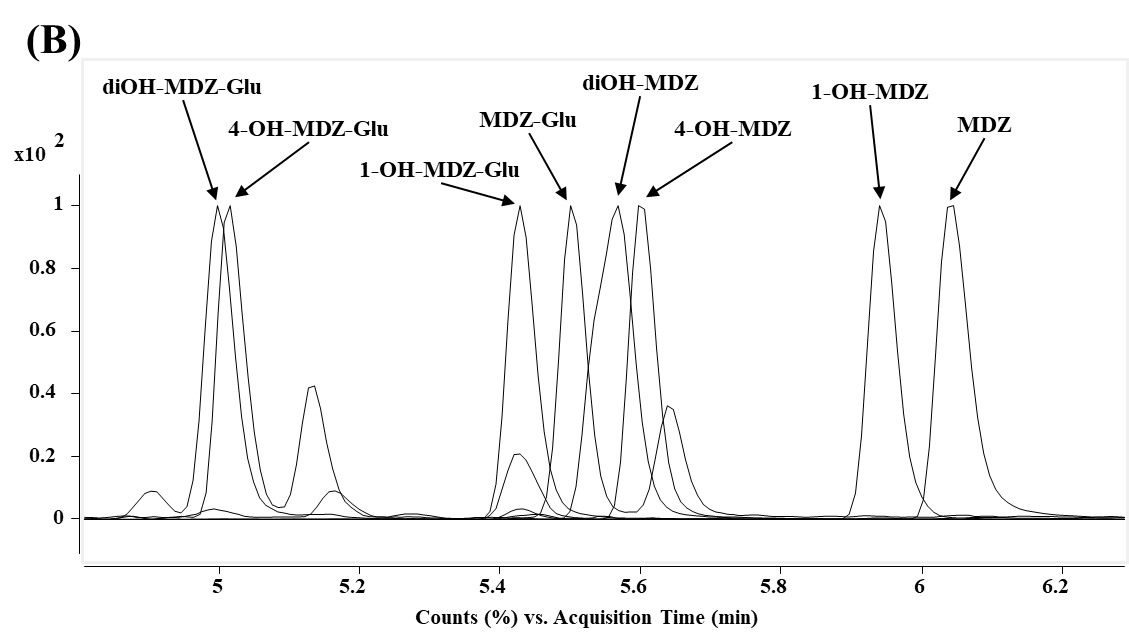
**

**Figure S4.** (A) Individual MRM chromatogram, (B) overlaid chromatogram of midazolam and each metabolite.

**Table S2.** Linearity, dynamic range, coefficient of determination, low limit of detection (LLOD), and limit of quantitation (LLOQ) of midazolam, 1-hydroxymidazolam, and 4-hydroxymidazolam

| **Analytes** | **Range (ng/mL)** | **Regression equation** | **r^2^** | **LLOD**  **(pg/mL)** | **LLOQ**  **(pg/mL)** |
| --- | --- | --- | --- | --- | --- |
| Midazolam | 0.10–5.00 | y = 70392x - 1449.8 | 0.9996 | 30 | 100 |
|  | 5.00–100.00 | y = 58981x + 108587 | 0.9996 |  |  |
| 1-OH-Midazolam | 0.025–5.00 | y = 26877x +1799.4 | 0.9997 | 25 | 75 |
|  | 5.00–100.00 | y = 20372x + 56463 | 0.9996 |  |  |
| 4-OH-Midazolam | 0.025–0.25 | y = 28315x + 248.77 | 0.9965 | 25 | 75 |
|  | 0.25–10.0 | y = 22722x + 3743.2 | 0.9991 |  |  |

**Table S3.** Recovery of midazolam in human serum and CSF during sample preparation

| **Sample** | **Concentration**  **(ng/mL)** | **Recovery (%)** | **Average ± STD (CV%)** |
| --- | --- | --- | --- |
| Serum 1 | 35.3 |  |  |
| Serum 2 | 32.6 |  |  |
| Serum 3 | 33.1 |  |  |
| Serum 1 + 35 ng/mL | 68.4 | 94.6 | 100.3 ± 5.0 (5.0%) |
| Serum 2 + 35 ng/mL | 68.9 | 103.5 |  |
| Serum 3 + 35 ng/mL | 69.1 | 102.9 |  |
| CSF 1 | 0.7 |  |  |
| CSF 2 | 0.7 |  |  |
| CSF 3 | 0.8 |  |  |
| CSF 1 + 35 ng/mL | 34.5 | 96.6 | 96.4 ± 1.7 (1.8%) |
| CSF 2 + 35 ng/mL | 33.8 | 94.6 |  |
| CSF 3 + 35 ng/mL | 35.1 | 98.0 |  |


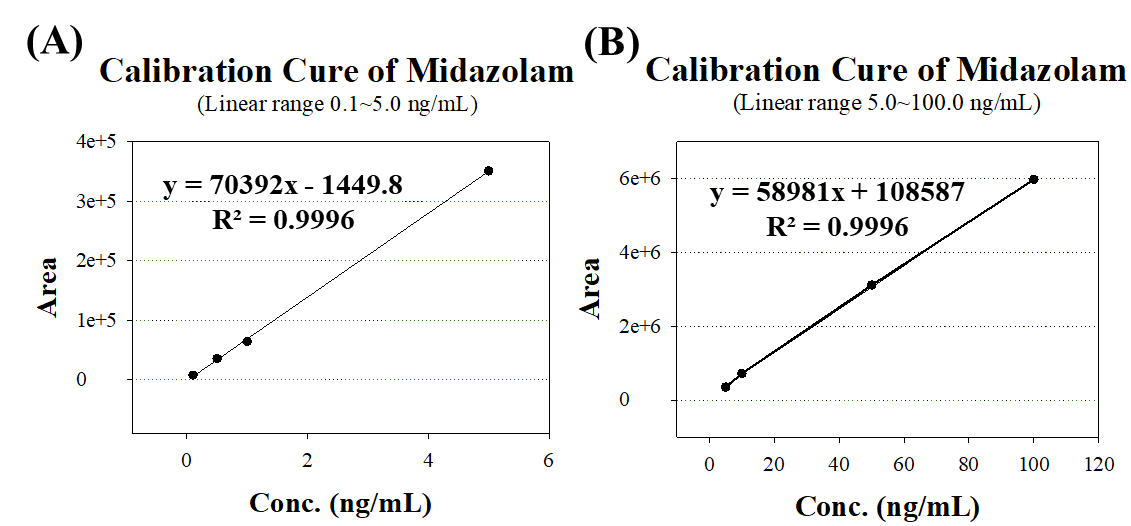


**Figure S5.** Calibration curve of midazolam with range of (A) 0.1 – 5.0 ng/mL, (B) 5.0 – 100.0 ng/mL

**eReferences**

1. Wessels, A. M. A., Bolhuis, M. S., Bult, W., Nijsten, M. W. N., Kneyber, M. C. J., & Touw, D. J.. A fast and simple method for the simultaneous analysis of midazolam, 1-hydroxymidazolam, 4-hydroxymidazolam and 1-hydroxymidazolam glucuronide in human serum, plasma and urine. *J. Chromatogr. B. Analyt. Technol. Biomed. Life. Sci.* **1162**, 122476 (2021).

2. Moorthy, G. S., Jogiraju, H., Vedar, C., Zuppa, A. F. Development and validation of a sensitive assay for analysis of midazolam, free and conjugated 1-hydroxymidazolam and 4-hydroxymidazolam in pediatric plasma: Application to Pediatric Pharmacokinetic Study. *J. Chromatogr. B. Analyt. Technol. Biomed. Life. Sci*. **1067**, 1-9 (2017).

3. Sano, T. et al. Sensitive determination of midazolam and identification of its two metabolites in human body fluids by column-switching capillary high-performance liquid chromatography/fast atom bombardment–mass spectrometry. *Leg. Med.* **3**, 149-156 (2001).

4. Burhenne, J. et al. Quantification of femtomolar concentrations of the CYP3A substrate midazolam and its main metabolite 1'-hydroxymidazolam in human plasma using ultra performance liquid chromatography coupled to tandem mass spectrometry. *Anal. Bioanal. Chem.* **402**, 2439-2450 (2012)
